# Supplementary material for: RNA Complexes with Nicks and Gaps: Thermodynamic and Kinetic Effects of Coaxial Stacking and Dangling Ends
Source: J Am Chem Soc. 2024 Jun 21;146(26):18083–94. doi: 10.1021/jacs.4c05115 (PMC11229006; doi:10.1021/jacs.4c05115)
Supplement: Supplementary file 1 — ja4c05115_si_001.pdf [file ja4c05115_si_001.pdf]

SUPPORTING INFORMATION

RNA complexes with nicks and gaps: thermodynamic and kinetic effects of coaxial stacking and dangling ends

Marco Todisco[1,†], Aleksandar Radakovic[1,2], and Jack W. Szostak[1,*]

[1]Howard Hughes Medical Institute, Department of Chemistry, The University of Chicago, Chicago, Illinois 60637, United States, [2]Department of Genetics, Harvard Medical School; Boston, Massachusetts 02115, United States

* Email: jwszostak@uchicago.edu, Tel: +1-617-943-9583.

Table of Contents

1. List of sequences used in this work 2
2. Comparison between analysis methods 3
3. Studying deviation from two-state behavior 6
4. Thermodynamic parameters for upstream nicks and gaps 7
5. Kinetic effect of upstream nicks and gaps 9
6. Dinucleotide binding 11
7. Predicting binding strength of chemically activated species 12
8. Disentangling hydrogen bond and stacking contributions to the formation of the double helix 13
9. Bibliography 16

1. List of sequences used in this work

The list of oligonucleotides used in this work is provided in Supporting Table 1. Nucleotides N1, N2 (and their complementary nucleotides N4, N3) have been systematically swapped to cover all canonical base-pairs at the interfaces of nicks and gaps. A sketch for the experimental sequences is provided in Supporting Figure S1.

Supporting Table 1. Sequences used in this work.

Strand type	Sequence	Notes
O1	5′p-**N**$_1$C(2Ap)CGAU-3′	
O2	5′-AUCGUG**N**$_4$-3′	
O3	5′-AUCGUG**N**$_4$**N**$_3$CUGGUACGUGCGACGU-3′	
O4	5′-ACGUCGCACGUACCAG**N**$_2$-3′	
O5	5′-ACGUCGCACGUACCA-3′	
O2	5′-AUCGUG**UG**C-3′	For kinetic study of dangling ends
O2	5′-AUCGUG**UG**CAAAAAAAAAAAAAAA-3′	For kinetic study of polyA tail
M1	5′-ACUGACCUACGUUGCU**CA**UCGUG**AU**CUGGUACGUGCGACGU-3′	For additivity study
M2	5′-ACGUCGCACGUACCAG**A**-3′	For additivity study
M3	5′p-**G**AGCAACGUAGGUCAGU-3′	For additivity study

Nucleotides in red highlight positions at nicked or gapped interfaces.

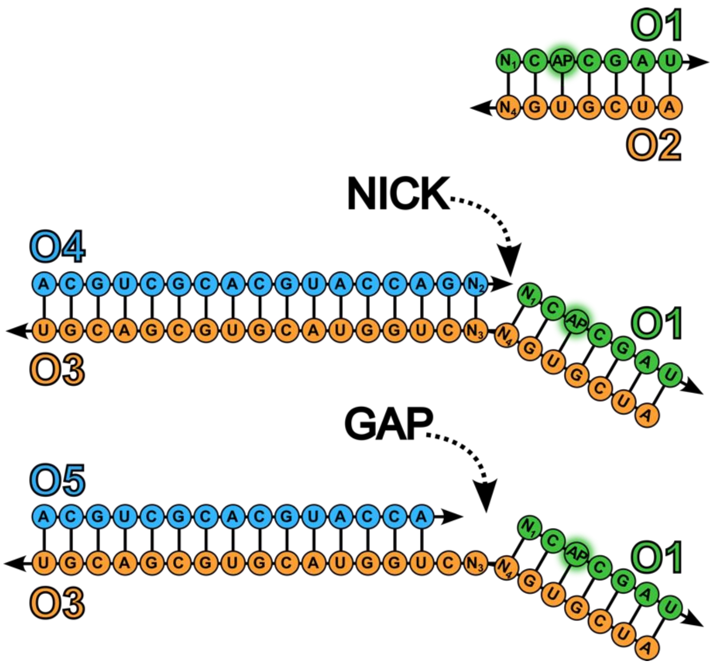

Supporting Figure S1. Sketch for the sequences used in our experimental design.

2. Comparison between analysis methods

In this work, we have characterized the thermodynamics of nicked and gapped oligonucleotide complexes of, with two alternative methods. With one method, each melting experiment was individually fitted, and the coefficients obtained this way were averaged across multiple experiments at different concentrations. Alternatively, the melting temperatures from all experiments were fitted as a function of oligonucleotide concentrations to obtain a second estimate of thermodynamic parameters. Under a two-state description, the values obtained with the two methods should be in agreement.

However, the values for ΔH° (Supporting Figure S2A) and ΔS° (Supporting Figure S2B) for the two methods are poorly correlated and widely spread. On the contrary, the values for extrapolated ΔG° at 37°C (Supporting Figure S2C) are very close (median absolute difference = 0.08 kcal/mol) except for nicked AC complex ($\Delta\Delta G°_{37°C}$ = 0.87 kcal/mol).

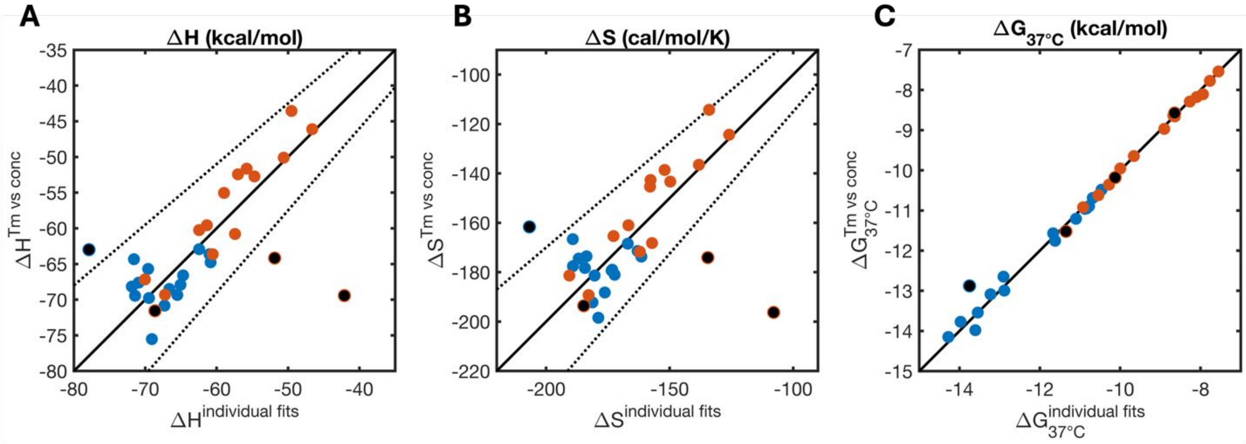

Supporting Figure S2. Difference in thermodynamic parameters estimated with two alternative methods as described in the main text. Blue dots referred to nicked complexes, red dots refer to gapped complexes. Black dots refer to anomalous samples that do not follow two-state behavior. Dotted lines show 15% interval bands, considered as a good threshold for establishing adherence to two-state behavior[1,2].

This counterintuitive result derives from compensatory effects in the differences in ΔH and ΔS as immediately visible comparing Supporting Figure S2A and Supporting Figure S2B. The two graphs look extremely similar, so that when the difference is taken to compute ΔG, the discrepancies become extremely small. The further away from the melting temperature at which the thermodynamic parameters have been estimated, the larger the discrepancies between the two methods. We investigated how big of a discrepancy between predictions from the two methods we should expect in a practical range of concentrations and temperatures, as depicted in Supporting Figure S3.

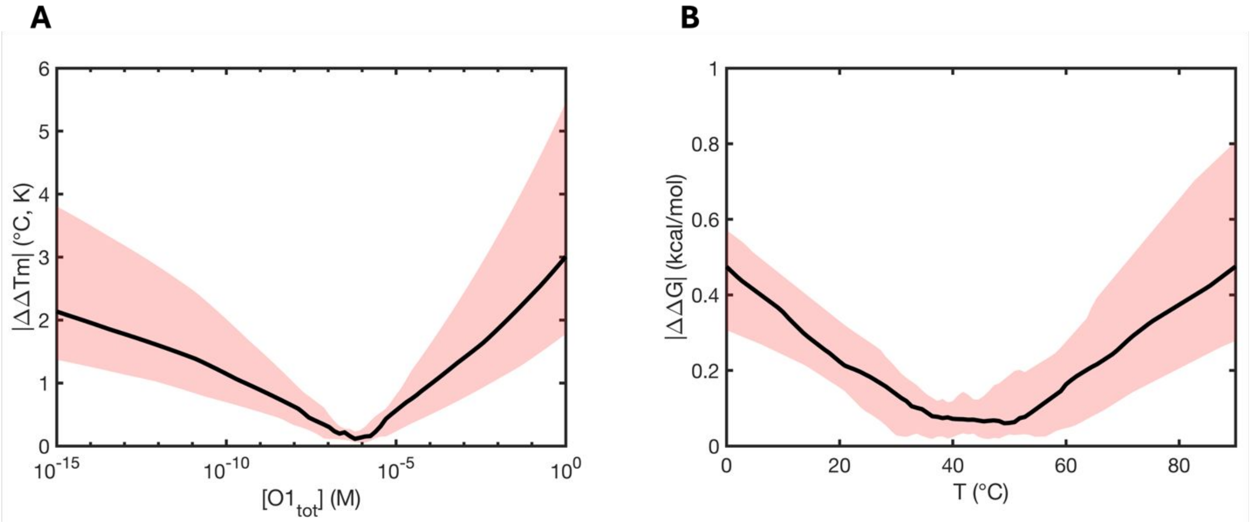

Supporting Figure S3. Discrepancies in predictions from the two analysis methods. **(A)** Absolute differences in predicted melting temperatures for our oligonucleotides using the two alternative methods in a practical range of concentrations. **(B)** Absolute differences in extrapolated ΔG° as a function of concentration for the two analysis methods. Black lines show median values, shaded areas envelope first and third quartiles for both studies.

We found that discrepancies in extrapolated melting temperatures in a wide range of practical concentrations do not typically vary more than a few degrees Celsius. Similarly, differences in extrapolated ΔG° are typically below 1 kcal/mol. The minimum in Supporting Figure S3A aligns with the typical working concentration of this work, while the minimum in Supporting Figure S3B aligns with the typical melting temperatures at our working concentrations, so that in both cases the two methods converge to the same values.
These simple considerations clearly highlight how directly comparing ΔH° and ΔS° values derived from a limited number of melting experiments can lead to very different conclusions depending on the specific analysis approach picked by the user, and thus in this work we focus on discussing impact of nicks and gaps in terms of ΔG°.
Finally, we can compare the values estimated with the two methods for blunt-ended duplexes to the ones computed with tabulated NN values (Supporting Table 2). By doing so, we found that analyzing our traces individually and averaging the fitted coefficients at different O1 concentrations provide values closest to those reported in the NNDB[3], and it is thus our preferred method to report our results for consistency with literature data. The only exception in the context of this work is to be found in the length dependence study, since the lower baseline for tetramers and pentamers could not be reliable estimated given the limitation of our temperature control (lower accessible temperature = -8.0 °C) and we had to rely on Tm vs conc.

Supporting Table 2. Thermodynamics of blunt ended duplexes

	NN		This work, individual fits				This work, Tm vs conc			
	ΔH°	ΔS°	ΔH°	ΔS°	|ΔΔH°|	|ΔΔS°|	ΔH°	ΔS°	|ΔΔH°|	|ΔΔS°|
**BLUNT, N1=A**	-54.65	-152.31	-52.93	-146.13	1.72	6.18	-47.10	-126.98	7.55	19.15
**BLUNT, N1=C**	-60.36	-165.98	-64.32	-178.26	3.96	12.28	-56.69	-153.76	3.67	24.51
**BLUNT, N1=G**	-61.85	-170.27	-60.13	-163.92	1.72	6.35	-53.35	-142.24	8.50	21.68
**BLUNT, N1=U**	-55.69	-155.31	-56.17	-157.46	0.48	2.15	-56.16	-157.36	0.47	0.10
**mean**					**1.97**	**6.74**			**5.05**	**16.36**

Comparison of thermodynamic parameters as determined through the two methods described in this work and compared with reference tabulated NN values for the calculation of blunt ended duplexes thermodynamic features. Values deriving from individual fits are more consistent with predicted features.

3. Studying deviation from two-state behavior

The work here presented relies on the assumption that the melting behavior is well described as a two-state transition. We found that this is not true for three case studies involving gapped complexes produced by systematically altering the identity of N1, N2 (and their complementary N4, N3) and for a single case study involving one AC nick. We investigated whether this behavior could be due to self-pairing of two O3:O4 (or O3:O5) duplexes due to their readily available dangling ends. Simple analysis through NUPACK confirms our hypothesis for gapped sequences, but not the nicked sequence (Supporting Figure S4).

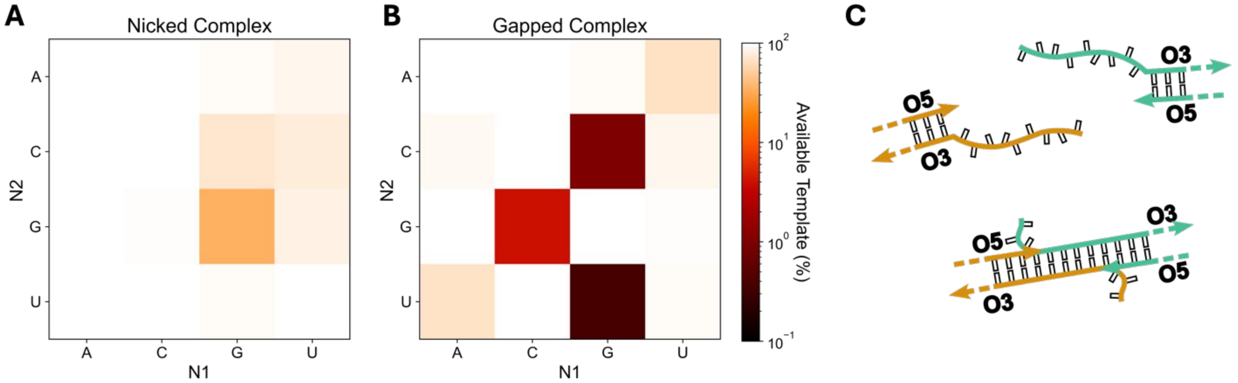

Supporting Figure S4. Self-interaction and deviation from two-state behavior. Available template as a function of N1, N2 sequence identity for (**A**) Nicked complexes (O3:O4 duplex), and (**B**) Gapped complexes (O3:O5 duplex). (**C**) CG, GC and UG in Gapped Complexes show signs of strong interaction leading to deviations from the expected two-state transition due to self-interactions. No self-interaction is expected for AC Nicked Complex.

To further test the self-pairing hypothesis, we analyzed the hybridization kinetics: if O3:O4 (or O3:O5) are self-paired, the outcome of the experiment will effectively be due to a relatively slow strand displacement process, and not the much faster conventional nucleation-zipper phenomenon.

Indeed, we find that the three gapped sequences here discussed exhibit an apparent hybridization rate slower by two (GC, $k_{on} \approx 2 \times 10^5\ M^{-1}s^{-1}$), three (CG, $k_{on} \approx 1 \times 10^4\ M^{-1}s^{-1}$) or even four orders of magnitude (UG, $k_{on} \approx 6 \times 10^3\ M^{-1}s^{-1}$) with respect to their relative blunt-ended duplex control (Supporting Table 5).

Once again, AC nicked duplexes do not show signs of self-interaction, with AC gapped duplex behaving in line with other nicked complexes ($k_{on} \approx 5 \times 10^6\ M^{-1}s^{-1}$, Supporting Table 5), supporting the hypothesis that the deviation from ideal two-state behavior is indeed a feature of this specific nicked interface.

4. Thermodynamic parameters for upstream nicks and gaps

In this work we have characterized the thermodynamic effect of an upstream nick or dinucleotide gap on a hybridizing RNA oligonucleotide. Here we report the full dataset of parameters. We have highlighted in red those cases that did not follow a clear two-state behavior and should thus be treated carefully.

Supporting Table 3. Thermodynamics of upstream nicks (coaxial stacking)

N2|N1	ΔH° individual fits (kcal/mol)	$\Delta S°_{37°C}$ individual fits (kcal/mol)	$\Delta G°_{37°C}$ individual fits (kcal/mol)	ΔH° Tm vs conc (kcal/mol)	$\Delta S°_{37°C}$ Tm vs conc (kcal/mol)	$\Delta G°_{37°C}$ Tm vs conc (kcal/mol)	$\Delta G°_{37°C}$ (kcal/mol) NNDB*
A|A	-14.37 ± 1.87	-35.07 ± 5.70	-3.49 ± 0.14	-23.72 ± 1.87	-65.24 ± 5.70	-3.49 ± 0.14	-0.93 ± 0.03
A|C	-13.56 ± 9.24	-28.50 ± 28.32	-4.72 ± 0.58	-6.31 ± 9.24	-7.86 ± 28.32	-3.87 ± 0.58	-2.24 ± 0.06
A|G	-9.34 ± 5.55	-16.41 ± 16.83	-4.25 ± 0.38	-16.43 ± 5.55	-39.08 ± 16.83	-4.31 ± 0.38	-2.08 ± 0.06
A|U	-8.53 ± 5.10	-16.06 ± 16.43	-3.55 ± 0.14	-10.43 ± 5.10	-21.99 ± 16.43	-3.61 ± 0.14	-1.10 ± 0.08
C|A	-7.91 ± 2.29	-15.30 ± 6.98	-3.17 ± 0.16	-17.65 ± 2.29	-46.68 ± 6.98	-3.18 ± 0.16	-2.11 ± 0.07
C|C	-4.75 ± 5.51	-0.58 ± 17.61	-4.57 ± 0.20	-18.82 ± 5.51	-44.65 ± 17.61	-4.97 ± 0.20	-3.26 ± 0.07
C|G	-11.45 ± 4.29	-25.28 ± 13.71	-3.61 ± 0.14	-10.98 ± 4.29	-24.41 ± 13.71	-3.41 ± 0.14	-2.36 ± 0.09
C|U	-9.33 ± 5.70	-18.78 ± 18.28	-3.51 ± 0.12	-13.17 ± 5.70	-30.88 ± 18.28	-3.60 ± 0.12	-2.08 ± 0.06
G|A	-12.12 ± 3.23	-26.14 ± 10.00	-4.01 ± 0.17	-20.81 ± 3.23	-54.06 ± 10.00	-4.04 ± 0.17	-2.35 ± 0.06
G|C	-6.61 ± 8.33	-5.38 ± 25.87	-4.94 ± 0.42	-10.92 ± 8.33	-19.83 ± 25.87	-4.77 ± 0.42	-3.42 ± 0.08
G|G	-11.29 ± 5.37	-20.35 ± 16.07	-4.98 ± 0.45	-16.10 ± 5.37	-36.05 ± 16.07	-4.91 ± 0.45	-3.26 ± 0.07
G|U	-13.42 ± 6.21	-29.29 ± 19.62	-4.33 ± 0.22	-9.53 ± 6.21	-17.14 ± 19.62	-4.21 ± 0.22	-2.24 ± 0.06
U|A	-9.53 ± 3.35	-20.81 ± 10.45	-3.07 ± 0.13	-15.84 ± 3.35	-41.48 ± 10.45	-2.97 ± 0.13	-1.33 ± 0.09
U|C	-7.56 ± 8.01	-10.85 ± 25.28	-4.20 ± 0.28	-11.47 ± 8.01	-23.81 ± 25.28	-4.08 ± 0.28	-2.35 ± 0.06
U|G	-6.48 ± 2.49	-9.31 ± 7.87	-3.59 ± 0.15	-15.15 ± 2.49	-36.74 ± 7.87	-3.76 ± 0.15	-2.11 ± 0.07
U|U	-4.84 ± 5.90	-5.49 ± 19.13	-3.13 ± 0.09	-7.48 ± 5.90	-14.03 ± 19.13	-3.13 ± 0.09	-0.93 ± 0.03

* Values for helix propagation.

Supporting Table 4. Thermodynamics of upstream gaps (dangling ends)

N2|N1	ΔH° individual fits (kcal/mol)	$\Delta S^{\circ}_{37°C}$ individual fits (kcal/mol)	$\Delta G^{\circ}_{37°C}$ individual fits (kcal/mol)	ΔH° Tm vs conc (kcal/mol)	$\Delta S^{\circ}_{37°C}$ Tm vs conc (kcal/mol)	$\Delta G^{\circ}_{37°C}$ Tm vs conc (kcal/mol)	$\Delta G^{\circ}_{37°C}$ (kcal/mol) NNDB*
A|A	-4.08 ± 1.56	-11.61 ± 4.84	-0.48 ± 0.12	-5.33 ± 1.56	-15.68 ± 4.84	-0.46 ± 0.12	-0.10
A|C	2.96 ± 5.62	11.56 ± 17.98	-0.63 ± 0.17	-2.89 ± 5.62	-7.24 ± 17.98	-0.64 ± 0.17	-0.60
A|G	-0.40 ± 2.50	1.88 ± 8.08	-0.99 ± 0.14	-10.27 ± 2.50	-29.51 ± 8.08	-1.12 ± 0.14	-1.20
A|U	5.57 ± 3.54	19.34 ± 11.64	-0.43 ± 0.08	6.06 ± 3.54	20.88 ± 11.64	-0.42 ± 0.08	-0.60
C|A	-9.53 ± 5.97	-26.55 ± 19.70	-1.29 ± 0.19	-13.15 ± 5.97	-38.36 ± 19.70	-1.25 ± 0.19	-0.70
C|C	-5.72 ± 7.44	-12.34 ± 23.77	-1.89 ± 0.19	-10.47 ± 7.44	-27.59 ± 23.77	-1.91 ± 0.19	-1.30
C|G	-8.51 ± 11.55	-20.82 ± 36.55	-2.06 ± 0.28	-18.20 ± 11.55	-51.33 ± 36.55	-2.29 ± 0.28	-1.70
C|U	-1.24 ± 3.47	0.33 ± 11.42	-1.34 ± 0.09	-4.63 ± 3.47	-10.81 ± 11.42	-1.28 ± 0.09	-0.80
G|A	3.42 ± 2.92	12.09 ± 9.26	-0.33 ± 0.10	3.56 ± 2.92	12.73 ± 9.26	-0.39 ± 0.10	-0.10
G|C	22.21 ± 5.68	70.35 ± 18.13	0.39 ± 0.33	-12.75 ± 5.68	-42.49 ± 18.13	0.43 ± 0.33	-0.40
G|G	1.16 ± 2.21	6.01 ± 7.32	-0.70 ± 0.16	-1.68 ± 2.21	-3.12 ± 7.32	-0.72 ± 0.16	-0.80
G|U	9.56 ± 3.94	31.54 ± 12.90	-0.22 ± 0.08	10.05 ± 3.94	33.00 ± 12.90	-0.19 ± 0.08	-0.50
U|A	-2.85 ± 2.32	-5.88 ± 7.28	-1.03 ± 0.11	-4.55 ± 2.32	-11.62 ± 7.28	-0.94 ± 0.11	-0.70
U|C	-2.91 ± 8.24	-4.51 ± 26.21	-1.51 ± 0.20	-12.62 ± 8.24	-35.50 ± 26.21	-1.61 ± 0.20	-1.10
U|G	8.27 ± 11.97	29.35 ± 38.00	-0.83 ± 0.38	-10.81 ± 11.97	-31.82 ± 38.00	-0.94 ± 0.38	-1.70
U|U	1.47 ± 3.63	7.73 ± 11.88	-0.93 ± 0.08	3.43 ± 3.63	14.07 ± 11.88	-0.93 ± 0.08	-0.80

* Values for 3′ dangling end stabilization. No associated uncertainties are available.

5. Kinetic effect of upstream nicks and gaps

In this work we have characterized the kinetic effect of an upstream nick or dinucleotide gap on a hybridizing RNA oligonucleotide. Here we report the full dataset of parameters. We have highlighted in red those cases that did not follow a clear two-state behavior. For gapped sequences, this is understood as due to self-pairing of the O3:O5 complexes as discussed in Supporting Data 2. The kinetic rates for such cases have to be interpreted as strand displacement rates and not hybridization rates.

It is important to notice how the deviation from two-state behavior does not create any anomaly in the hybridization rate of C|A, supporting the hypothesis that such deviation is indeed an intrinsic property of that nicked interface.

We have highlighted in light blue the two control experiments (compare with Blunt, N1 = A) for the effect of 3′ a dinucleotide dangling end and an extra polyA$_{15}$ tail.

Supporting Table 5. Kinetics of upstream nicks and gaps

Experiment (N2|N1)	k_{on} ($M^{-1}s^{-1}$)	k_{off} (s^{-1})
**Blunt, N1 = A**	$(1.97 \pm 0.31) \times 10^{7}$	$(8.54^{+0.59}_{-0.55}) \times 10^{-1}$
**Blunt, N1 = C**	$(1.49 \pm 0.09) \times 10^{7}$	$(3.87^{+1.18}_{-0.91}) \times 10^{-2}$
**Blunt, N1 = G**	$(1.96 \pm 0.15) \times 10^{7}$	$(4.00^{+0.82}_{-0.68}) \times 10^{-2}$
**Blunt, N1 = U**	$(0.91 \pm 0.20) \times 10^{7}$	$(4.47^{+0.50}_{-0.45}) \times 10^{-1}$
**gapped A|A**	$(0.49 \pm 0.08) \times 10^{7}$	$(6.09^{+0.41}_{-0.39}) \times 10^{-2}$
**gapped A|C**	$(0.73 \pm 0.08) \times 10^{7}$	$(5.40^{+0.77}_{-0.68}) \times 10^{-3}$
**gapped A|G**	$(0.63 \pm 0.13) \times 10^{7}$	$(7.93^{+1.49}_{-1.25}) \times 10^{-4}$
**gapped A|U**	$(0.36 \pm 0.08) \times 10^{7}$	$(1.23^{+0.07}_{-0.07}) \times 10^{-1}$
**gapped C|A**	$(0.35 \pm 0.09) \times 10^{7}$	$(5.85^{+0.95}_{-0.82}) \times 10^{-3}$
**gapped C|C**	$(0.38 \pm 0.07) \times 10^{7}$	$(1.37^{+0.61}_{-0.42}) \times 10^{-4}$
**gapped C|G**	$(1.13 \pm 0.11) \times 10^{4}$	$(1.02^{+0.32}_{-0.24}) \times 10^{-7}$
**gapped C|U**	$(3.03 \pm 0.38) \times 10^{6}$	$(8.12^{+0.45}_{-0.43}) \times 10^{-3}$
**gapped G|A**	$(0.64 \pm 0.03) \times 10^{7}$	$(2.05^{+0.08}_{-0.08}) \times 10^{-1}$
**gapped G|C**	$(2.05 \pm 0.48) \times 10^{5}$	$(2.70^{+0.89}_{-0.67}) \times 10^{-4}$
**gapped G|G**	$(1.14 \pm 0.18) \times 10^{7}$	$(6.47^{+1.87}_{-1.45}) \times 10^{-3}$
**gapped G|U**	$(0.45 \pm 0.05) \times 10^{7}$	$(4.29^{+0.21}_{-0.20}) \times 10^{-1}$
**gapped U|A**	$(0.47 \pm 0.08) \times 10^{7}$	$(4.26^{+0.76}_{-0.64}) \times 10^{-2}$
**gapped U|C**	$(0.39 \pm 0.04) \times 10^{7}$	$(2.16^{+0.60}_{-0.47}) \times 10^{-4}$
**gapped U|G**	$(0.57 \pm 0.92) \times 10^{3}$	$(9.14^{+28.68}_{-6.93}) \times 10^{-8}$
**gapped U|U**	$(0.56 \pm 0.05) \times 10^{7}$	$(5.60^{+0.33}_{-0.31}) \times 10^{-2}$

**nicked A|A**	$(0.37 \pm 0.09) \times 10^{7}$	$(1.28^{+1.16}_{-0.61}) \times 10^{-4}$
**nicked A|C**	$(0.50 \pm 0.10) \times 10^{7}$	$(1.27^{+0.73}_{-0.46}) \times 10^{-5}$
**nicked A|G**	$(0.90 \pm 0.15) \times 10^{7}$	$(4.23^{+5.14}_{-2.32}) \times 10^{-6}$
**nicked A|U**	$(1.82 \pm 0.31) \times 10^{6}$	$(2.96^{+2.14}_{-1.24}) \times 10^{-5}$
**nicked C|A**	$(0.57 \pm 0.12) \times 10^{7}$	$(6.23^{+3.94}_{-2.42}) \times 10^{-4}$
**nicked C|C**	$(0.67 \pm 0.10) \times 10^{7}$	$(7.75^{+4.46}_{-2.75}) \times 10^{-7}$
**nicked C|G**	$(0.47 \pm 0.05) \times 10^{7}$	$(1.36^{+0.30}_{-0.24}) \times 10^{-5}$
**nicked C|U**	$(2.96 \pm 0.80) \times 10^{6}$	$(8.31^{+0.89}_{-0.81}) \times 10^{-5}$
**nicked G|A**	$(0.89 \pm 0.19) \times 10^{7}$	$(1.36^{+0.69}_{-0.46}) \times 10^{-4}$
**nicked G|C**	$(0.68 \pm 0.01) \times 10^{7}$	$(2.46^{+2.97}_{-1.34}) \times 10^{-6}$
**nicked G|G**	$(1.14 \pm 0.19) \times 10^{7}$	$(1.47^{+1.60}_{-0.76}) \times 10^{-6}$
**nicked G|U**	$(0.33 \pm 0.05) \times 10^{7}$	$(4.19^{+0.53}_{-0.47}) \times 10^{-5}$
**nicked U|A**	$(0.67 \pm 0.17) \times 10^{7}$	$(9.83^{+4.88}_{-3.26}) \times 10^{-4}$
**nicked U|C**	$(1.00 \pm 0.10) \times 10^{7}$	$(1.71^{+0.63}_{-0.46}) \times 10^{-5}$
**nicked U|G**	$(0.95 \pm 0.15) \times 10^{7}$	$(3.61^{+3.23}_{-1.71}) \times 10^{-6}$
**nicked U|U**	$(0.66 \pm 0.10) \times 10^{7}$	$(6.82^{+1.28}_{-1.08}) \times 10^{-4}$
**dangling C|A, polyA$_{15}$ tail**	$(1.13 \pm 0.08) \times 10^{7}$	NA
**dangling C|A**	$(1.93 \pm 0.15) \times 10^{7}$	NA

6. Dinucleotide binding

Given the importance of short oligonucleotides in the context of the origin of life, and particularly of imidazolium bridged dinucleotides[4], we measured the binding energy of two canonical dinucleotides to templates containing 2-aminopurine (Supporting Figure S5A) at room temperature.

To perform these experiments, RNA dinucleotides of sequence 5′-GU-3′ and 5′-UG-3′ were hybridized to a dinucleotide gap and the fluorescent emission of 2-aminopurine was monitored over time. The quenching was fitted with a simple hyperbolic binding curve having amplitude and binding constant as free parameters, yielding K equal to 411 μM and 104 μM for 5′-GU-3′ and 5′-UG-3′ respectively (Supporting Figure S5B).

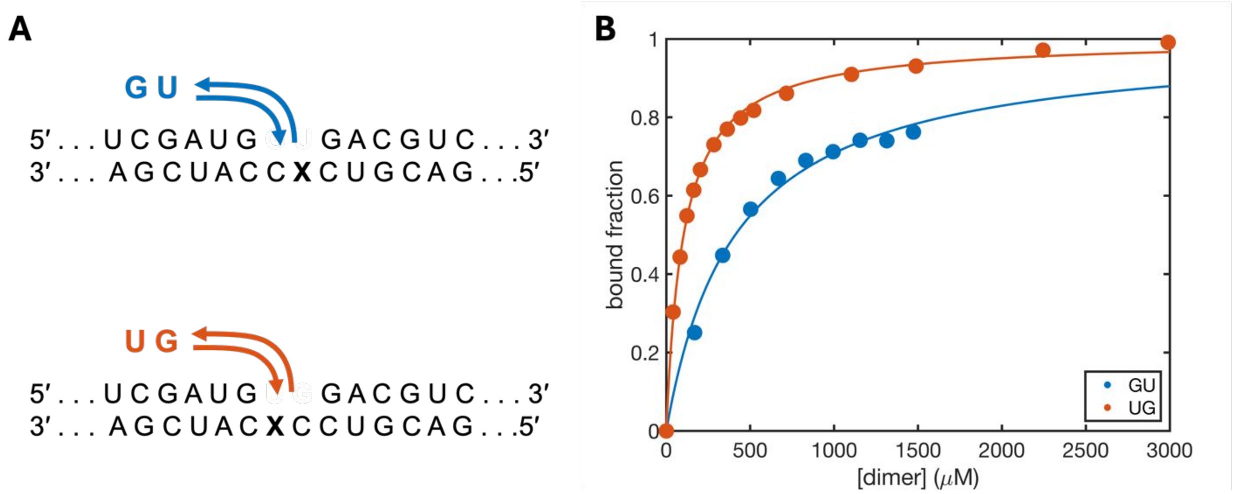

Supporting Figure S5. Dinucleotide binding. (**A**) Sketch for experimental design used to measure dinucleotides binding to a dinucleotide gap. The X in the template sequence stands for 2-aminopurine. (**B**) Normalized binding curves as determined through quenching experiments for GU and UG dimers.

The values obtained with predictions using our experimentally determined values for coaxial stacking (see main text) yield $\Delta G°_{20°C}$ for 5′-GU-3′ equal to -6.1 kcal/mol and 5′-UG-3′ equal to -7.9 kcal/mol, to be compared with values obtained here equal to -4.5 kcal/mol and -5.3 kcal/mol respectively. The offsets are equal to -1.6 kcal/mol and -2.6 kcal/mol, a difference that can be rationalized as coming from the length dependence discussed in the main text and potentially from an imperfect additivity of the two coaxial interfaces next to the extremely short dinucleotides.

Comparing the binding constants determined this way with K_M values from primer extension experiments from literature in a buffer with comparable ionic strength[5], we found our dissociation constants for 5′-GU-3′ and 5′-UG-3′ to be more stable, with ΔΔG° at 20°C equal to 1.9 kcal/mol and 1.2 kcal/mol respectively. We attribute these differences to the presence of the chemically activating imidazolium bridge in the dinucleotides used by Ding et al.[4]

7. Predicting binding strength of chemically activated species

Binding strengths of ultrashort (1 ~ 4 nt) chemically activated oligonucleotides are analyzed in this work. The complete dataset of experimentally determined values from literature[6,5,7] and predictions is presented in Supporting Table 6.

Supporting Table 6. Predictions for binding strength of chemically activated species

Species	Experimental System	$\Delta G°_{20°C}$ (kcal/mol) Predictions, new coaxial stacking	$\Delta G°_{20°C}$ (kcal/mol) Predictions, coaxial stacking from NNDBΩ	$\Delta G°_{20°C}$ (kcal/mol) Experiment
A*CGC	Upstream Nick	-8.34	-7.48	-5.96
A*CG	Upstream Nick	-4.29	-3.43	-3.65
A*C	Upstream Nick	-1.48	-0.62	-1.74
*A	Upstream Nick	-1.57	-0.71	-1.12
*CUGA	Upstream Nick	-7.90	-7.13	-7.07
*CGC	Upstream Nick	-7.38	-6.61	-7.03
A*A	Double Nick	-2.17	+1.37	-4.28
A*C	Double Nick	-3.30	-0.52	-3.25
A*G	Double Nick	-5.29	-2.22	-5.55
A*U	Double Nick	-1.19	+1.55	-2.25
C*A	Double Nick	-4.69	-1.72	-4.83
C*C	Double Nick	-5.58	-3.37	-4.56
C*G	Double Nick	-6.76	-4.27	-6.07
C*U	Double Nick	-3.38	-1.22	-2.49
G*A	Double Nick	-5.32	-1.82	-5.13
G*C	Double Nick	-6.11	-3.37	-3.83
G*G	Double Nick	-8.06	-5.04	-6.91
G*U	Double Nick	-3.88	-1.18	-2.63
U*A	Double Nick	-2.97	+1.11	-3.42
U*C	Double Nick	-3.84	-0.52	-2.37
U*G	Double Nick	-5.69	-2.08	-4.15
U*U	Double Nick	-1.27	+2.01	-1.70

8. Disentangling hydrogen bond and stacking contributions to the formation of the double helix

To evaluate the effect of hydrogen bonds and coaxial stacking in the transition from single stranded RNA to double stranded RNA, we employed the approaches from Frank-Kamenetskii[8] and from Zacharias[9].
Following the approach from Frank-Kamenetskii, the single stranded RNAs are treated as completely unstacked at equilibrium (Supporting Figure S6A). The free energy change for the formation of a double helix as quantified from the NNDB must thus be equal to the straightforward sum of the free energy changes due to (i) stacking all bases and (ii) hydrogen bonding them. Importantly, this model implies that stacking the single stranded RNAs in solution prior to their hybridization is not an energetically favored process (higher free energy state).
The model from Zacharias on the contrary treats single stranded RNAs as partially pre-stacked in solution. The free energy change for the formation of a double helix as quantified from the NNDB must thus be mostly due to hydrogen bonding (Supporting Figure S6B). Counterintuitively, since the single stranded RNAs are at their stacking free energy minimum, stacking the bases that were not previously stacked in the single-stranded state leads to a positive free energy contribution (penalty).

The mathematical treatment of the model is more complicated than the one from Frank-Kamenetskii since it requires quantifying the extent of pre-stacking for the single-stranded RNAs. Briefly, the free energy change associated with the stacking of a fraction *x* of bases is:

$$\Delta G(x) = \Delta G^{stacking} + RTln\left(\frac{x}{1-x}\right)$$

For convenience, Zacharias computes the integral to define a Free Energy function along the *x* coordinates,

$$G(x) = x\Delta G^{stacking} + RT(1-x)ln(1-x) + RTxln(x) + C$$

and uses this to calculate the Free Energy function associated with helix propagation by subtracting the constant term *C* and the NN energy, so that *G(x)* has its minimum along *x* corresponding to the fraction of stacked bases at equilibrium, and it is offset by the ΔG from the NNDB. For example, for the formation of a CG pair,

$$G(x) = x\Delta G_{CG}^{stacking} + 2RT(1-x)ln(1-x) + 2RTxln(x) - G_{min} - \Delta G_{CG}^{NN,helix\,propagation}$$

where $\Delta G^{stacking}$ is the energy associated with coaxial stacking as determined in this work.
This model produces the free energy functions shown in Supporting Figure S6B. Given the large energies determined in this work, almost all nucleobases are expected to be stacked at equilibrium (mean $x \approx 0.96$), with helix propagation driven by hydrogen bonds.
In this model, the total free energy change due to hydrogen bonding two nucleobases is the value of *G(x)* for $x = 1$ and the free energy penalty for stacking the previously unstacked bases is equal to $G(1) - \Delta G_{CG}^{NN,helix\,propagation}$.

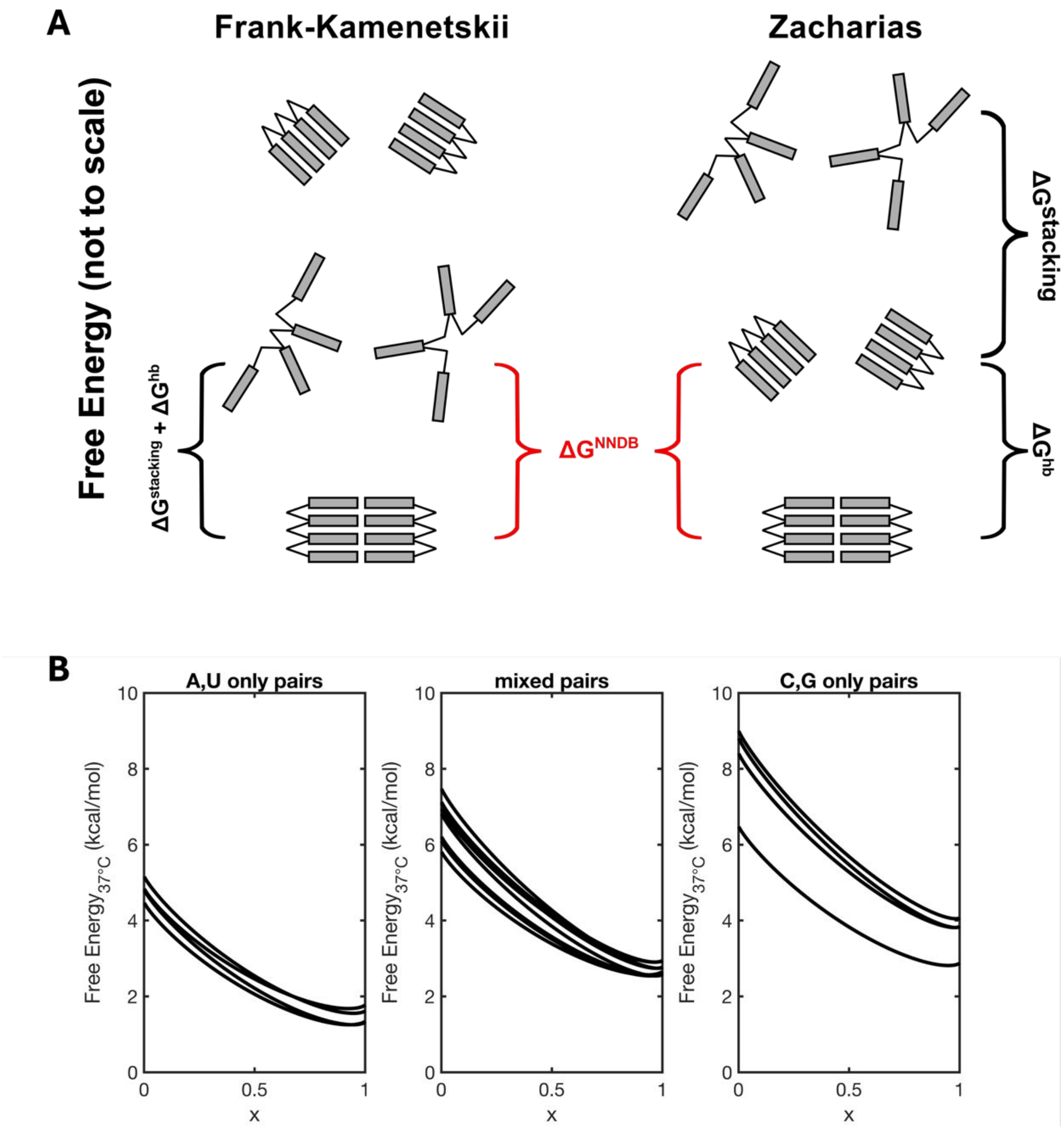

Supporting Figure S6. A) Free energy diagram for RNA hybridization according to Frank-Kamenetskii (left) and Zacharias (right). Sketches readapted from Zacharias (2020)[9]. In the first case, the most stable state for single stranded RNA is unstacked, and the free energy change for transitioning to a double helix is the sum of free energy change for stacking all bases and hydrogen bonding them. In the second case the single stranded oligonucleotides are more stable as pre-stacked in solution with a free energy change dependent on the stacking energy of the nucleobases (the example shows the extreme case where they are fully stacked). The free energy change for transitioning to a double helix is mostly due to hydrogen bonding of pre-stacked single stranded RNA. **B)** Free energy profile for the formation of a new base pair as a function of the fraction of stacked nucleobases (x). The position of the minimum along the x axis corresponds to expected fraction of stacked nucleobases (in single stranded RNA) at equilibrium as derived from the experimentally measured coaxial stacking. The Free Energy value at such a minimum corresponds to the value associated with the formation of a given base pair from the NNDB as described in text.

9. Bibliography

(1) Xia, T.; SantaLucia, J.; Burkard, M. E.; Kierzek, R.; Schroeder, S. J.; Jiao, X.; Cox, C.; Turner, D. H. Thermodynamic Parameters for an Expanded Nearest-Neighbor Model for Formation of RNA Duplexes with Watson−Crick Base Pairs. *Biochemistry* **1998**, *37* (42), 14719–14735. https://doi.org/10.1021/bi9809425.

(2) Sieg, J. P.; Arteaga, S. J.; Znosko, B. M.; Bevilacqua, P. C. MeltR Software Provides Facile Determination of Nucleic Acid Thermodynamics. *Biophysical Reports* **2023**, *3* (2), 100101. https://doi.org/10.1016/j.bpr.2023.100101.

(3) Turner, D. H.; Mathews, D. H. NNDB: The Nearest Neighbor Parameter Database for Predicting Stability of Nucleic Acid Secondary Structure. *Nucleic Acids Research* **2010**, *38* (suppl_1), D280–D282. https://doi.org/10.1093/nar/gkp892.

(4) Walton, T.; Szostak, J. W. A Highly Reactive Imidazolium-Bridged Dinucleotide Intermediate in Nonenzymatic RNA Primer Extension. *J. Am. Chem. Soc.* **2016**, *138* (36), 11996–12002. https://doi.org/10.1021/jacs.6b07977.

(5) Ding, D.; Zhou, L.; Giurgiu, C.; Szostak, J. W. Kinetic Explanations for the Sequence Biases Observed in the Nonenzymatic Copying of RNA Templates. *Nucleic Acids Res* **2022**, *50* (1), 35–45. https://doi.org/10.1093/nar/gkab1202.

(6) Zhou, L.; O'Flaherty, D. K.; Szostak, J. W. Template-Directed Copying of RNA by Non-enzymatic Ligation. *Angew. Chem. Int. Ed.* **2020**, *59* (36), 15682–15687. https://doi.org/10.1002/anie.202004934.

(7) Ding, D.; Zhang, S. J.; Szostak, J. W. Enhanced Nonenzymatic RNA Copying with *in-Situ* Activation of Short Oligonucleotides. *Nucleic Acids Research* **2023**, *51* (13), 6528–6539. https://doi.org/10.1093/nar/gkad439.

(8) Yakovchuk, P. Base-Stacking and Base-Pairing Contributions into Thermal Stability of the DNA Double Helix. *Nucleic Acids Research* **2006**, *34* (2), 564–574. https://doi.org/10.1093/nar/gkj454.

(9) Zacharias, M. Base-Pairing and Base-Stacking Contributions to Double-Stranded DNA Formation. *J. Phys. Chem. B* **2020**, *124* (46), 10345–10352. https://doi.org/10.1021/acs.jpcb.0c07670.
